# Spatial Ecology of the Palm-Leaf Skeletonizer, *Homaledra sabelella* (Lepidoptera: Coleophoridae)

**DOI:** 10.1371/journal.pone.0022331

**Published:** 2011-07-22

**Authors:** James T. Cronin

**Affiliations:** Department of Biological Sciences, Louisiana State University, Baton Rouge, Louisiana, United States of America; Umea University, Sweden

## Abstract

Understanding the processes that determine the distribution of populations is a fundamental goal in ecology. In this study, I determined the relative contribution of space and the biotic and abiotic environment to the distribution of the palm-leaf skeletonizer *Homaledra sabalella* (PLS; Lepidoptera: Coleophoridae) among patchily distributed dwarf palmettos (*Sabal minor*; Arecaceae). Based on surveys conducted at two sites in the Sherburne Wildlife Management Area, Louisiana, I found that the distribution of the PLS was primarily related to local environmental conditions – number of PLS increased with palmetto height, was greater in dry versus wet habitats, and varied in an inconsistent way with the type of understory cover. Spatial structure of the forest and isolation of the host plant were of minor importance to the distribution of the PLS. Based on a series of experiments, the mechanisms underlying the effects of these environmental variables on PLS abundance were elucidated. Tall palmettos have a greater abundance of PLS because they are 2.5 times more likely to be colonized than small palmettos. Tall palmettos do not represent better hosts (in terms of PLS survival to pupation, pupal length, or risk of parasitism). Similarly, an open understory increased colonization by two-fold, relative to a shrub understory, but understory type had no effect on host quality. Wet soils greatly reduced palmetto quality as a host (survival and pupal length), but only for the smallest palmettos (<0.75 m height). Finally, corroborating the survey data, my dispersal experiment revealed that the PLS is a strong flier and that local PLS populations (i.e., infested palmettos) are likely well connected by dispersal. I conclude by discussing how landscape-level changes at Sherburne Wildlife Management Area, owing to recent hurricane activity, could affect the risk of palmetto infestation by the PLS.

## Introduction

A fundamental goal in ecology is to understand the processes determining the abundance of a species across space [1], [2], [3], [4]. Historically, distributional patterns were thought to be niche based; i.e., related primarily to local environmental conditions [1], [5], [6]. Later, island biogeography and metapopulation theories emphasized the primacy of geography (i.e., the size and isolation of suitable habitat patches), and more recently, the field of landscape ecology broadened the scope to include other spatial components of the environment (e.g., matrix composition, presence of habitat edges, proportional abundance of different habitat types) in determining the distribution of a population [7], [8], [9]. Regardless of the factors involved, dispersal limitation may induce additional spatial heterogeneity in the distribution of a species (e.g., [10], [11], [12]. Although there have been numerous studies on the subject, the relative contributions of local environmental conditions and space to the distributions of species varies considerably (see [13]). Moreover, recent studies suggest that the contribution of each factor to the distribution of a species can vary with spatial scale [12], [14]. Consequently, there is no shortcut to understanding the processes influencing the distribution of a focal species – targeted studies are essential.

In attempting to determine the relative contributions of environmental response variables and spatial heterogeneity to species distributions, ecologists have had to contend with the possibility that these two variables are seriously confounded [2], [4], [15], [16]. This confounding can be caused by the response variables (i.e., species abundances) being spatially autocorrelated, or dependent on explanatory variables that are themselves spatially autocorrelated. If unaccounted for, autocorrelation can greatly bias interpretation of statistical models that test for relationships between species abundance and environmental response variables. Recently, a variety of methods been developed to partition the pure spatial effects from the environmental effects on species abundance – e.g., principle coordinates of neighbor matrices and spatial eigenvector mapping [2], [4], [16], [17]. A growing list of studies have utilized this methodology to partition spatial and environmental effects on species distributions (e.g., [18], [19], [20], [21], [22]; see also [13]). Although these methods have proven insightful, under certain circumstances, particularly when the spatial scale chosen is too crude, they can lead to spurious conclusions [23]. It is therefore essential that the spatial scale of the study be chosen properly and/or that experiments be conducted to confirm cause-and-effect relationships between environmental variables and species distributions. Unfortunately, this type of supporting information is relatively rare [23].

In this study, I determined the relative contributions of space and the local environment to the distribution of the palm-leaf skeletonizer (*Homaledra sabalella* Chambers; Lepidoptera: Coleophoridae) among patchily distributed dwarf palmettos (*Sabal minor*; Arecaceae) in south-central Louisiana, U.S.A. The palm leaf skeletonizer (PLS) is considered a pest of various palm species [24], [25] but to date, almost nothing is known about the factors influencing its spatial distribution among palms at the local or regional scale. I conducted a survey of PLS abundance among dwarf palmettos in two localities and then used spatial eigenvector mapping (SEVM) to determine the relative contributions of space and the environment to their distributional patterns. Next, I conducted a series of experiments to determine how specific environmental factors deemed important in the analysis of survey data (palmetto height, understory cover, soil moisture) affect PLS colonization of host plants, juvenile performance, and parasitism. Finally, I evaluated the importance of dispersal limitation as a potential driver of spatial patterning in the PLS. These results are used to evaluate how landscape change can affect the risk of palmetto infestation by the PLS.

## Materials and Methods

### Natural history

*Sabal minor* is one of four species of extant palmettos native to North America [26]. It is an abundant southeastern species ranging from southern Florida to coastal North Carolina and west to eastern Oklahoma and eastern Texas [26], [27]. Dwarf palmetto grows most commonly in floodplains, alluvial forests, and moist beach habitats but can also be found in mesic and drier forests and prairie habitats [28]. Although it can reach a height of 5 m [26], [28], in Louisiana, heights rarely exceed 3–4 m (pers. obs.).

The PLS has been reported throughout Florida and the western Caribbean [24], [29], [30] but has recently been discovered elsewhere along the Gulf Coast (Mississippi, Louisiana) ([31], Cronin pers. observ.). Within this range, the PLS is a specialist of native and several introduced palm species [30], [32], [33]. Caterpillars feed in colonies under a dense protective cover of silk. Damage is easily visible and can span the entire underside of the frond. Pupation also occurs beneath the cover within silken tubes [32]. Adults are short lived, with males and females surviving for up to four and 20 days, respectively [32]. In the Florida panhandle to Louisiana, five generations or more can occur per year ([25], Cronin unpubl. data).

### Field survey of PLS

**Figure 1:**
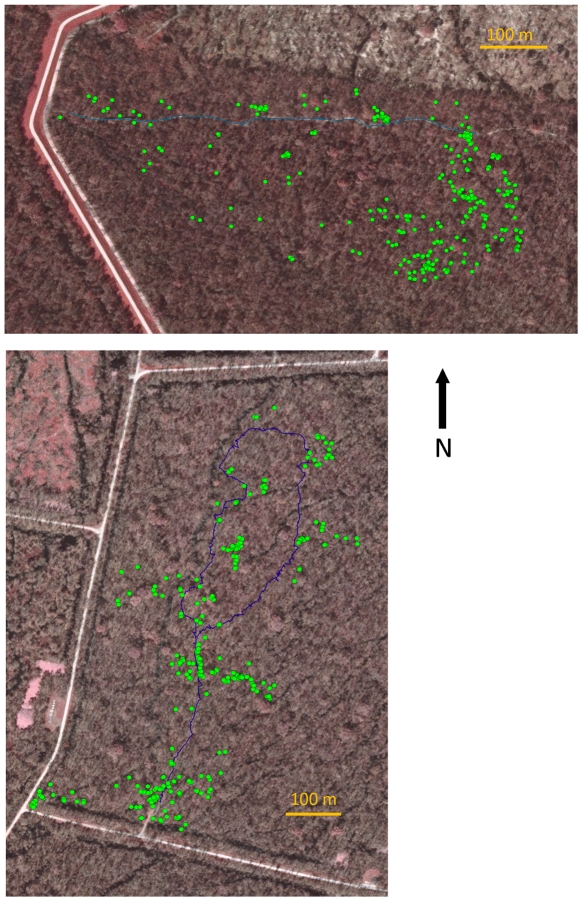
Research sites ATV Trail (ATV) and Nature Trail (NAT) in the Sherburne Wildlife Management Area, Louisiana. The locations of palmettos are marked in green.

In February of 2006, I discovered a PLS outbreak on *S. minor* at Sherburne Wildlife Management Area (WMA) in south-central Louisiana. Research was conducted at two sites, Nature Trail (NAT) and ATV Trail (ATV) (see File S1 for details). At each site, I mapped the distribution of all palmettos >0.25 m in height using a Trimble GeoXT GPS with Hurricane external antenna (precision≤0.5 m). NAT was 36 ha and had 237 palmettos and ATV was 27 ha and had 319 palmettos (Fig. 1).

In May, 2006, at a time when the PLS was predominantly in the late pupal stage, I conducted a survey of the palmettos at each site. Palmetto height (ground to tip of tallest frond; measured in 0.5 m increments), number of green and open fronds, and number of infested fronds were recorded. It had rained heavily the week prior to the survey and I recorded soil wetness immediately adjacent to the palmetto (wet = standing water, damp = wet soil but no standing water, dry). In addition, I classified understory cover within 2 m of the palmetto as either predominately inhabited by ferns (*Pteridium aquilinum*), dense shrubs, other palmettos, or bare ground. Infested portions of the fronds were clipped with scissors and the material placed in a Ziploc bag and stored on ice. A separate bag was used for each frond.

For each palmetto, I computed an index of isolation (*I*) that was a negative-exponential function of the proximity of a focal infestation to all other infested palmettos within a site (see File S1). As *I* increases, isolation of the PLS population on a palmetto increases. This isolation index is widely used in the metapopulation literature [34], [35], [36], [37].

In the laboratory, the Ziploc bags with infested portions of fronds were hung from a rack until adult moths and parasitoids had completed emergence. At that time, the PLS pupal cases were removed from their silken cases and dissected. For each pupa, I recorded its fate: emerged, parasitized, or dead from unknown causes (<2% fell into the latter category).

### Distribution of palmettos

The spatial dispersion of a plant species can strongly influence the pattern of distribution of its herbivores. To determine whether palmettos at each site had a uniform, random or clumped distribution, I computed Morisita's index of dispersion, *I_d_* [38] (see File S1). If *I_d_*<0, the distribution was uniform, equal to zero it was random, and greater than zero it was clumped.

### PLS spatial patterns

The goal with the survey data was to determine the relative contribution of spatial structure (location or isolation) and the local environment (palmetto height, soil wetness, understory cover) to PLS abundance per palmetto. Various modeling approaches can be used to account for spatial autocorrelation in PLS distributions [4], [16], [17]. Here, I used spatial eigenvector mapping (SEVM) to encapsulate the spatial variation in the distribution of palmettos as a set of eigenvectors (i.e., spatial filters) that can be treated as independent variables in subsequent models for predicting PLR abundance [4], [15] (see File S1 for details). A generalized linear mixed model was used to assess the effects of the following categorical environmental variables on PLS number per palmetto: palmetto understory cover (bare, ferns, shrubs, other palmettos), soil wetness (dry, damp, wet), height (0–0.5 m, 0.5–1.5 m, 1.5–2.5 m, 2.5–3.5 m, >3.5 m) and isolation (low [*I_d_*≤0.1], medium [0.1<*I_d_*<1], high [*I_d_*>1]) (see File S1). All spatial filters (eigenvectors) and spatial coordinates (easting and northing) were treated as continuous dependent variables in the model. Finally, all first-order interactions were also included in the model.

The relative importance of each source of variation to predicting PLS abundance was evaluated using McFadden's pseudo-*R* ^2^ (*R* ^2^ *_M_*; [39]). This pseudo *R* ^2^ is an approximate measure of goodness of fit for general linear models and can be used to estimate the percent contribution of a particular subset of model predictors to the goodness of fit ( = %*R* ^2^ *_M_*). For more details, see File S1.

### Environmental determinants of PLS spatial structure

Results from the previous survey revealed that PLS abundance was significantly related to palmetto height, soil wetness and understory cover (see Results). In 2007, I conducted two experiments to understand the mechanistic basis for these findings. In particular, the experiments were designed to determine whether these environmental variables affected PLS abundance by promoting differential colonization of, or larval/pupal performance on, palmettos.

In the first experiment, I tested whether the colonization of uninfested palmettos was dependent on palmetto height or understory cover. In late February, 2007, an equal number of small (0.50–0.75 m tall) and large (1.5–2.0 m tall) uninfested palmettos (*n* = 120 total) were transferred in pots to the ATV site. Potted palmettos were placed around the perimeters of five naturally occurring palmetto clusters that were infested with overwintering PLS (i.e., source patches). Each source patch was embedded within a matrix comprised of a mosaic of shrubs and bare ground. The experimental design involved placing potted palmettos 3 m from the source perimeter in either a pure shrub or pure open matrix (equal numbers in each matrix type). Additional details regarding the experimental design are in File S1.

In early June, at which time the second generation of the year was in the late larval-pupal stages, I recorded the number of active, distinct PLS colonies (colonies with green silken covers, separated by ≥10 cm) on the potted palmettos. After adult emergence, the infested portions of the fronds were removed, placed in Ziploc bags, and returned to the laboratory. The number of pupal cases was determined for each frond. The effect of palmetto height, understory and their interaction on colonization success was evaluated with logistic regression (see File S1 for details).

In the second experiment, I tested whether palmetto height, understory cover, and soil wetness affected PLS abundance by influencing larval performance and pupal parasitism. Proportion of caterpillars surviving to the pupal stage, pupal length (mm), and proportion of pupae surviving to adult eclosion were used as indices of performance. In September, 2007, at a time when new PLS colonies were forming, I selected uninfested, naturally occurring palmettos that fell into one of three height categories (small = 0.5–0.75 m, medium = 1.5–2 m, large = 3.0–4.0 m) and one of four understory/wetness categories (fern, shrub, open-dry and open-wet). I inoculated each palmetto with 18.4±2.2 early instar larvae by attaching excised portions of infested fronds to the fronds of the focal palmettos (Figure S1). For more details regarding this experimental design, see File S1.

Six weeks later, shortly after adult eclosion, fronds infested from the transfer of moths were collected and returned to the laboratory. Pupal cases were removed from under the silken cover and measured for total length using digital calipers. The fate of the PLS (dead, moth eclosed, parasitized) was determined. The effects of understory cover/wetness and palmetto height on number of PLS that achieved the pupal stage, pupal length (mean of all pupae per palmetto), and proportion of pupae parasitized was assessed with separate two-way fixed factor ANCOVAs (see File S1).

### Dispersal limitation

An important factor that may influence the distribution of a species, and in particular the degree of autocorrelation in space, is dispersal ability [10], [11], [12], [15]. Limited dispersal can cause positive autocorrelation and strong spatial structuring of the population. To evaluate whether dispersal limitation constrains the distribution of PLS within a site, I performed two releases of PLS adults at an uninfested forested site and monitored the appearance of new infestations on surrounding potted palmettos. Palmettos were positioned in concentric circles at distances of 10 m to 200 m from an experimentally created source population of PLS. The spatial extent of this study was chosen in light of the spatial dispersion of palmettos within ATV and NAT - the maximum nearest neighbor distance was only 79 m for ATV and 65 m for NAT. Therefore, I assumed that if dispersal limitation were important to the within-site distribution of PLS, we should see evidence of dispersal limitation at the scale of this study - three times the maximum nearest neighbor distance.

Three weeks after the experiment was initiated, I counted the number of new PLS colonies on each of the potted palmettos – an indication that the palmetto was successfully oviposited on by adult female moths emanating from the source. A second repetition of the experiment was performed one generation later. Additional details regarding this experiment can be found in File S1.

I evaluated the fit of two phenomenological dispersal models to the colonization-with-distance data, the negative exponential function (NEF) and the inverse power function (IPF) [40] (see File S1). Relative to a Gaussian function that emerges from simple diffusion models, the exponential and power distributions have fatter tails (indicative of long-distance dispersal). The tail in the power distribution is higher than for the exponential distribution [40], [41], [42]. Fat-tailed distributions are common in the literature [43], [44] including lepidopterans [42] and have important large-scale implications for the distribution of a species [45]. The fit of both models to the dispersal data were evaluated with least-squares regression (see File S1). From the best fit model, I estimated the median dispersal distance and the distance that contained 95% of the colonies and their 95% confidence intervals.

## Results

### Distribution of palmettos

Palmettos at both field sites had strongly clumped distributions (Fig. 1). At ATV, the variance-to-mean ratio was 28.0 and index of dispersion (*I_d_*) of 2.70. *I_d_* was significantly greater than zero (χ^2^ = 560.6, *df* = 21, *P*<0.001) indicating significant aggregation. Similarly, at NAT, the variance-to-mean ratio = 107.7 and *I_d_* = 6.48 (χ^2^ = 1324.4, *df* = 54, *P*<0.001). At ATV, 80% of the palmettos were ≤1.5 m in height (mean ± SE: 0.92±0.04 m) whereas at NAT, only 51% were ≤1.5 m tall (1.54±0.06 m).

### PLS spatial patterns

In May, 2006, 28.5% and 41.3% of all palmettos were infested with PLS at ATV and NAT, respectively. Numbers of moth pupae per palmetto ranged from 0 to 154 at ATV (mean ± SE: 6.8±1.1) and 0 to 340 at NAT (21.3±3.1) and the distribution of moth pupae per palmetto at each site was strongly right skewed. The abundances per palmetto at each site were significantly different based on a Mann-Whitney U test (χ^2^ = 14.1, *df* = 1, *P*<0.001). At both sites, the spatial eigenvector mapping (SEVM) approach and forward selection procedure indicated that there was no spatial autocorrelation in the data and that no eigenvectors were informative regarding spatial structure. Therefore, the analyses below did not include any of the eigenvectors as predictor variables.

**Figure 2:**
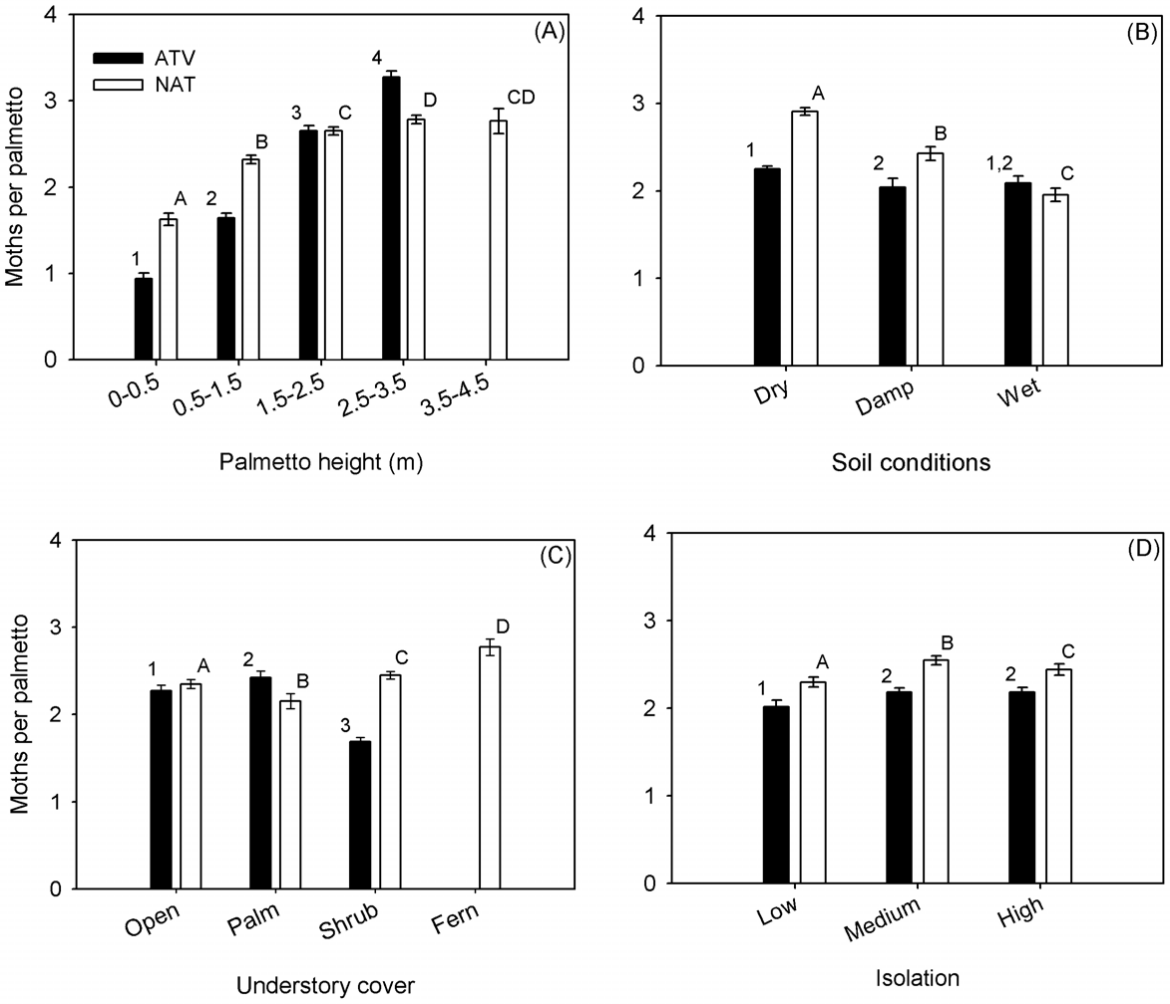
The influence of (A) palmetto height, (B) soil wetness (dry, damp and wet),(C) understory cover (open, other palmettos, shrubs or ferns), and (D) isolation (low, medium, high) on the mean number of PLS pupae per palmetto (± se). Separate Poisson-regression analyses were performed for ATV (open bars) and NAT (filled bars) and differences (*P*<0.05) among treatment means were denoted by different numbers (ATV) and letters (NAT).

The distribution of PLS pupae per palmetto was well explained by the full model containing all dependent variables and their first-order interactions. *R* ^2^ *_M_* was 0.49 and 0.54 for ATV and NAT respectively. For both sites, palmetto height contributed the most to the goodness of fit of this model. The percent reduction in *R* ^2^ *_M_* (%*R* ^2^ *_M_*) following the removal of height and all interactions involving height was 63% for ATV and 44% for NAT. In general, as height increased, PLS density increased (Fig. 2A; *F*_3,273_ = 401.3, *P*<0.001; NAT: *F*_4,175_ = 111.0, *P*<0.001). Among the main predictor variables for ATV, the ranked contribution to goodness of fit (i.e., %*R* ^2^ *_M_* following the removal of a predictor variable and all interaction involving it) was height (63%), understory (40%), isolation (31%), easting (24%), and wetness (12%). It should be noted here that these percentages are relative and not additive because comparisons are non orthogonal. In comparison, the rankings for NAT were height (44%), easting (26%), understory (22%), isolation (20%), northing (16%), and wetness (11%).

Palmettos in wetter soils tended to have lower densities of PLS than in drier soils (Fig. 2B; ATV: *F*_2,273_ = 20.3, *P*<0.001; NAT: *F*_2,175_ = 632.4, *P*<0.001). In NAT, where standing water persisted longer following storm activity, PLS abundance was 33% lower in wet as compared to dry soils. The effects of understory cover differed between sites. At ATV, PLS abundance was lowest when palmettos were surrounded by shrubs and highest when surrounded by other palmettos; a 30% difference (Fig. 2C; *F*_2,273_ = 44.2, *P*<0.001). In contrast, at NAT, PLS abundance was lowest in palmetto-dominated habitats and highest in fern-dominated habitats (Fig. 2C; 22% difference; *F*_3,175_ = 13.2, *P*<0.001). Although isolation effects were statistically significant (Fig. 2D; ATV: *F*_2,273_ = 36.4, *P*<0.001; NAT: *F*_2,175_ = 150.4, *P*<0.001), the expectation that abundance would decrease with increasing isolation was not upheld. PLS per palmettos was highest for the medium- and high-isolation categories at ATV and for the medium-isolation category at NAT. For both sites, there was only an 8–10% difference between the isolation categories with the highest and lowest abundance. Finally, at ATV, PLS abundance decreased by an estimated 11% from the eastern edge to western edge of the site (a span of 678 m; *F*_1,273_ = 28.7, *P*<0.001); and at NAT it increased by 12% from east to west (*F*_1,175_ = 29.74, *P*<0.001) and 8% from south to north (*F*_1,175_ = 14.6, *P*<0.001).

For both ATV and NAT, all first-order interactions were statistically significant (P<0.03). However, for approximately one-half of them, %*R* ^2^ *_M_* was <5% suggesting that each contributed very little to the goodness of fit of the overall model (Table S1). At both sites, palmetto height interacted with understory cover and soil wetness. In general, the positive relationship between palmetto height and PLS abundance (see Fig. 2A) was stronger for wet and shrubby habitats then dry and open habitats, but the contributions to goodness of fit were relatively small (%*R* ^2^ *_M_* between 5% and 10%; Table S1). The remaining interactions also tended to contribute relatively little to the model's goodness of fit and/or were relatively uninteresting (e.g., interactions between easting or northing positions and the environmental variables).

### Colonization of host plants

**Figure 3:**
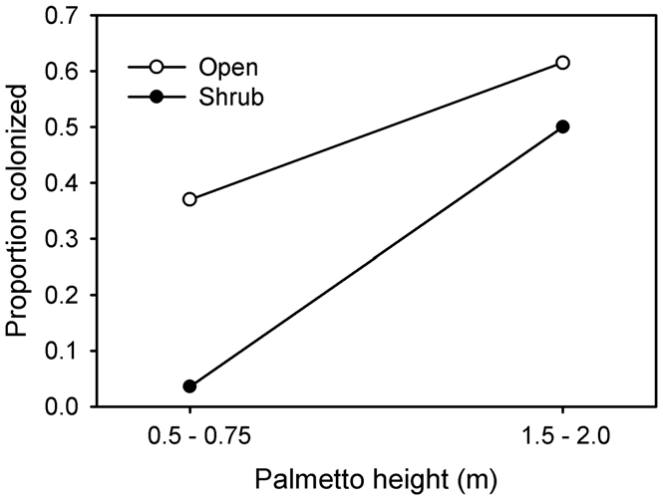
The effect of palmetto height and understory cover (open or shrub) on the proportion of potted palmettos bearing new PLS colonies.

Larger palmettos (1.5–2.0 m tall) were 2.5 times more likely to be colonized than smaller palmettos (<0.75 m tall) (54% vs. 20%; *F*_1,99_ = 13.2, *P*<0.001; Fig. 3). In addition, palmettos in a relatively bare matrix were twice as likely to be colonized as those in a shrub-inhabited matrix (49% vs. 24%; *F*_1,99_ = 6.8, *P* = 0.011; Fig. 3). Much of the understory effect was the result of small palmettos in a shrubby matrix having only a 4% probability of being colonized. This latter result was largely responsible for the nearly significant height-understory interaction (*F*_1,99_ = 3.8, *P* = 0.054). Source population had no significant effect on colonization success (*F*_1,99_ = 2.3, *P* = 0.070).

### PLS performance

**Figure 4:**
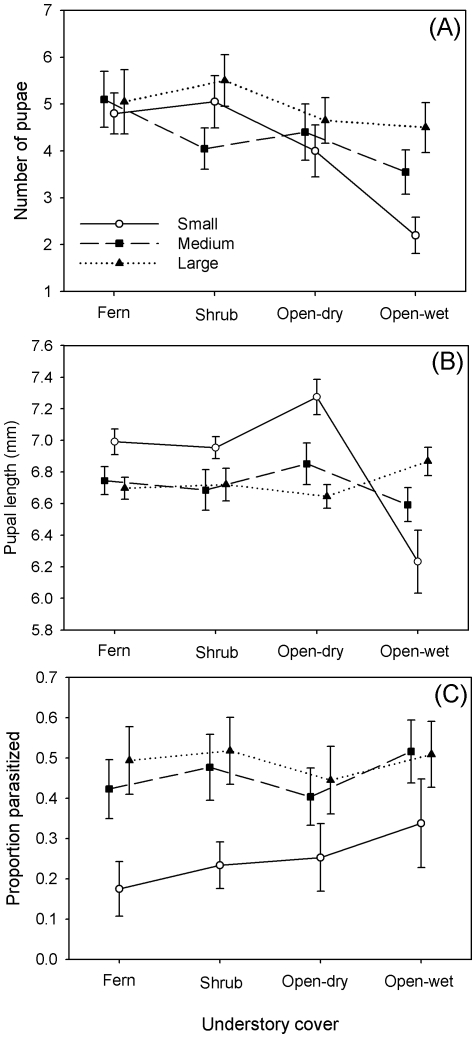
Performance of PLS juveniles on naturally occurring palmettos that differed in height (small = 0. 5–0.55 m, medium = 1.5–2 m, large = 3.0–4.0 m) and understory cover/wetness (fern, shrub, open-dry, and open-wet). (A) Number of PLS that developed to the pupal stage, (B) pupal length (mm), and (C) the proportion of pupae parasitized. All values are means ± se per palmetto.

Following the transfer of approximately 18 early instar PLS larvae to naturally occurring uninfested palmettos, only 4.4±0.2 (*n* = 240) reached pupation. The number of pupae per palmetto was strongly affected by understory type (*F*_3,228_ = 5.42, *P* = 0.001) but this was mostly influenced by the open-wet habitat (Fig. 4A). Palmettos in an open-wet understory had 28% fewer pupae than in any of the other understory types and this was mostly driven by the scarcity of pupae on small palmettos in the wet habitat. Among understory types, the difference in number of pupae was significant for the open-wet vs. fern (Tukey HSD, *P* = 0.002) and open-wet vs. shrub (*P* = 0.005) but not open-wet vs. open-dry (*P* = 0.140) comparisons. Pupal number decreased by 22% from the smallest to largest palmettos, but this trend was not significant using the Bonferroni-corrected α (α_adj_ = 0.0125) (*F*_2,228_ = 3.13, *P* = 0.046). There was no significant interaction of height and understory on number of pupae (*F*_2,228_ = 1.34, *P* = 0.238).

In comparison, the only factor that significantly affected pupal length was the interaction between palmetto height and understory (*F*_6,210_ = 3.30, *P* = 0.004). This result is most evident when examining the small palmettos. Whereas pupal length was lowest for palmettos in the open-wet understory, it averaged 14% higher for all other understory types (Fig. 4B). If the open-wet understory is excluded from the model, there is a highly significant effect of palmetto height on pupal length (*F*_2,161_ = 13.09, *P*<0.001). In this case, pupal length on the large and medium sized palmettos was significantly smaller than on the smaller palmettos (5.7% and 4.7%, respectively, Tukey's HSD, *P*<0.001). There was no difference between the two larger palmetto categories with regard to pupal length (*P* = 0.633).

Finally, the proportion of PLS pupae parasitized was only affected by palmetto height (*F*_2,210_ = 10.44, *P*<0.001) – pupae on the small palmettos suffered 45% and 50% less parasitism than the medium and large palmettos, respectively (Tukey's HSD, *P*≤0.001 for both comparisons; Fig. 4C). There was no evidence that parasitism was density dependent (*F*_1,210_ = 0.18, *P* = 0.67).

### Dispersal limitation

**Figure 5:**
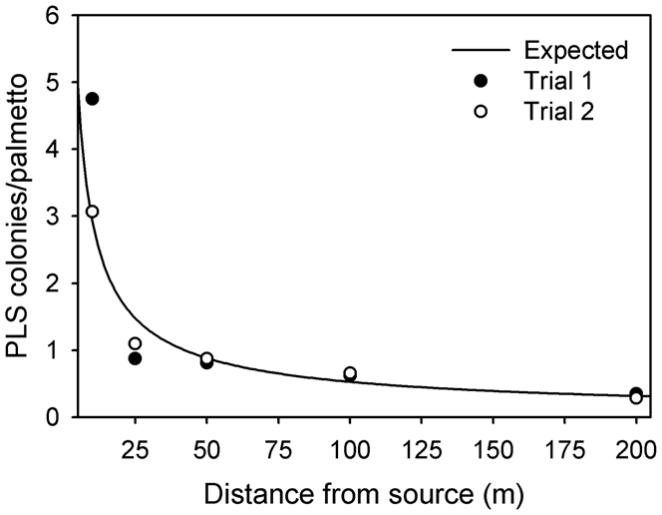
The relationship between distance from the source of PLS and the number of newly formed PLS colonies on potted palmettos. Data are reported for two trials and the curve is the expected number of colonies based on the inverse power function (*ln* colony number = −0.75*[*ln* distance]+2.79; *R* ^2^ = 0.90, P<0.001).

The appearance of new PLS colonies declined with distance from the experimentally created source population, but new colonies were evident at the farthest distance of 200 m (Fig. 5). Although the NEF provided an adequate fit to the data (*R* ^2^ = 0.70, *P* = 0.003), the IPF provided a much better fit to the data (Fig. 5, *R* ^2^ = 0.90, *P*<0.001). Based on the Monte Carlo simulation, the median dispersal distance was estimated to be 130.5 m (95% CIs: 106 m, 154 m) and 95% of the newly formed colonies were predicted to occur within 856 m (837 m, 874 m) of the source.

## Discussion

Based on an analysis of 158 published datasets, Cottenie [13] concluded that an average of 48% of the total variance in community structure was explained by the combined effects of space and the environment. Although the pure environmental effects had more explanatory power than the pure spatial effects, the difference was relatively small (22% and 16%, respectively). Moreover, the relative contributions of each factor varied considerably from dataset to dataset. In general, these findings argue against a purely neutral assembly of communities [10] and instead suggest that both environmental and spatial heterogeneity are important drivers of species distributions [46].

Even though the PLS is considered a pest species of palms [24], [25], almost nothing was known about the factors influencing its spatial distribution in natural or managed ecosystems. In this study, I found that aspects of the local environment, but not spatial structure, were significant determinants of PLS distributions among dwarf palmettos in Louisiana. The spatial eigenvector mapping (SEVM; [4]) approach indicated that no eigenvectors were informative regarding spatial structure indicating the absence of spatial autocorrelation among local populations of the PLS. This finding is similar to that of Sattler et al. [22] on the distribution of spiders, bees and birds in an urban landscape. In contrast, many other studies using SEVM or analogous methodologies have found a significant contribution of space to the distribution of focal species [18], [19], [20], [47]. In light of the study by Pinto & MacDougal [12], it must be acknowledged that the relative importance of space and the environment may change as spatial scale is increased.

The absence of a significant spatial component to the distribution of the PLS is likely due in part to the high dispersal ability of this moth species. In the two field sites, increased isolation of palmettos was not associated with a decrease in abundance of moths (Fig. 3D). Moreover, in the dispersal experiment, the appearance of new colonies with distance from the source was best fit by an inverse power function indicative of fat tails and a relatively high frequency of long-distance dispersers [40], [41], [42]. Fifty percent of the adult female moths dispersed at least 130 m and 5% dispersed at least 855 m. Relative to the maximum nearest-neighbor distance among palmettos (72 m), PLS appears quite vagile (at least within the context of my 27–36 ha field sites). Leptokurtic dispersal kernels, like the one found for the PLS are common in a wide diversity of taxa [43], [48], [49], [50], [51], including Lepidoptera [42], [52]. Finally, the high dispersal ability coupled with the absence of spatial structure argues against the possibility that the PLS exists as a metapopulation [8]. Instead, it suggests that the collection of infested palmettos within a forested site may better be described as one large patchy population [53].

The absence of spatial structure for the PLS within the two field sites could also be a consequence of neutral (e.g., non contagious) interactions with individuals of the same or other species [10], [15], [54], [55]. In a companion study that focused on the guild of parasitoids associated with the PLS pupal stage, I (unpubl. data) found that only one of three common species exhibited strong spatial structuring that could have affected the distribution of the PLS (the bethyliid, *Goniozus* sp.). However, the proportion of host parasitized by this species was relatively low and likely insufficient to affect PLS spatial patterns.

Local environmental variables were the primary drivers of PLS distributional patterns. These environmental variables were diverse and included an attribute associated with the host plant (plant height), soil conditions (wetness), and local landscape structure (understory cover). This finding supports the view that niche-based processes are critical to the distribution of species [6], [13], [56]. Palmetto height was the single most important environmental variable – as palmetto height increased, so did PLS density. Based on my field experiments, this height effect is not caused by larger palmettos being of higher quality for PLS development or providing a greater refuge from parasitism. On the contrary, if we exclude small palmettos in wet environments, PLS pupal length was significantly greater and the proportion of hosts parasitized was significantly lower on the smallest palmettos.

Small palmettos in relatively dry soils may represent a more nutritious resource to the PLS. Although, there is no published data to support this with the PLS, many plant species have higher nutrient and/or lower chemical defense levels when they are young or small [57], [58], [59], [60]. As a suitable patch for the PLS, smaller palmettos may represent a partial refuge from parasitism because they are less likely to be colonized by their parasitoids (Cronin, unpubl. data). Finally, small palmettos in wet soils appear to be very poor hosts. During the course of this study, low-lying areas had standing water for several days to a couple of weeks following moderate-to-heavy rains. In some cases, the lower half of the palmettos, including infested areas of fronds, were submerged. This was especially evident at NAT where standing water persisted much longer than at ATV (pers. observ.). Naturally occurring palmettos in wet soils had 33% and 7% fewer moths than dry soils at NAT and ATV, respectively. Moreover, the performance experiment demonstrated that small palmettos in wet soils had the lowest number of moths surviving to pupation and the smallest pupal size. Flooding may either directly influence the growth and survival of the moth or indirectly affect the moth by reducing palmetto quality as a host. Johnson [61] similarly found that flooding greatly reduced the survival of a rolled-leaf beetle and generated strong sink dynamics during the wet season.

The landscape variable, understory cover, was the second or third most important variable (depending on the site) affecting PLS abundance but its effects differed qualitatively between sites. At NAT, PLS per plant was highest for palmettos associated with a bracken fern understory cover, intermediate in an open or shrubby cover, and lowest under a canopy of other larger palmettos. At ATV, ferns were scarce, and PLS had the lowest abundance in a shrub cover, intermediate in the open and highest under other palmettos. Interestingly, the field experiments revealed very little effect of understory cover on number of PLS surviving to the pupal stage, pupal length or proportion of hosts parasitized. The exception involved the smaller palmettos in open-wet vs. open-dry habitats. In open-dry habitats, PLS achieved the greatest pupal length relative to all other understory covers, supporting the earlier argument that smaller palmettos unstressed by excess water are a high quality food source for the PLS. Although the experiment only focused on two understory covers (open and shrub) in the colonization experiment, it appears that PLS adults are much more capable of finding a host plant (regardless of size) when the palmettos are in open habitat.

The above findings support a growing body of literature on the importance of landscape structure to the spatial and temporal population dynamics of natural systems (for reviews see [62], [63], [64]). The composition of the matrix surrounding a patch is known to affect emigration and immigration rates [65], [66], [67], extinction risk [68], [69], [70], and source-sink and regional population dynamics [71], [72]. It is also the case that open matrix habitats can promote higher patch densities than more heavily vegetated matrices ([69]; but see [71]).

At both sites, there were significant directional trends in PLS abundance per palmetto. Abundance increased east to west at ATV but west to east at NAT. Moreover, at NAT, abundance increased from south to north. Owing to the differences between sites, these trends were not likely driven by prevailing winds. Although edge or road effects on species distributions are common [73], [74], [75], [76], there was no consistent association of PLS with the occurrence of roads (which occurs on the east side of both sites) or clear cuts (which was present on the north side of ATV). Additional research will be necessary to determine the cause of these directional trends in PLS abundance.

In summary, this research approach yields a clear picture of the spatial population structure of the PLS and the environmental factors that determine its distribution. At the scale of this study (27–36 ha), the local environment overrides space in its contribution to the distribution of the PLS. An understanding of the causes for the spatial distribution of the PLS can yield insights regarding other trophic levels associated with the PLS [2], [4], [13], [16]. At the basal trophic level, PLS can achieve density levels that can kill the palmetto [25], [32], especially when they are small (Cronin unpubl. data). However, early palmetto stages represent a partial refuge from PLS attack, even though it is a better host in general (in terms of body size and lower parasitism). In addition, a change in understory structure could greatly affect palmetto risk of attack (owing to altered colonization rates). For example, hurricane Gustav (August 31, 2008) caused extensive and prolonged flooding and much tree damage at Sherburne WMA. Three weeks of standing water over much of ATV and NAT may have contributed to the near disappearance of PLS seven months later (Cronin unpubl. data). Also, in the years following the hurricane, shrubs grew to dominate the wind thrown areas and overall, became much more prevalent at the two sites. Based on the findings in this study, a shift in landscape structure to one with a greater prevalence of shrubs is expected to reduce the likelihood of palmettos being colonized (no change expected in PLS growth and survival). The long-term effect of Gustav on the population dynamics of the PLS are currently under investigation.

## Supporting Information

Figure S1 **Photograph of experimentally established PLS colonies.** Paperclips were used to attach excised portions of infested palmetto fronds (containing early instar PLS) to uninfested fronds.
(PDF)

Table S1 Results from Poisson regression analyses for the effects of palmetto understory cover (bare, ferns, shrubs, other palmettos), soil wetness (dry, damp, wet), height (0–0.5 m, 0.5–1.5 m, 1.5–2.5 m, 2.5–3.5 m, >3.5 m) and isolation (low [*I_d_*≤0.1], medium [0.1<*I_d_*<1], high [*I_d_*>1]) and position (easting and northing) on the number of PLS pupae per palmetto.
(PDF)

File S1 Supplement for Materials and Methods section.
(PDF)
