# Supplementary material for: Spatial Ecology of the Palm-Leaf Skeletonizer, Homaledra sabelella (Lepidoptera: Coleophoridae)
Source: PLoS One. 2011 Jul 22;6(7):e22331. doi: 10.1371/journal.pone.0022331 (PMC3142117; doi:10.1371/journal.pone.0022331)

SUPPORTING INFORMATION

Figure S1. Photograph of experimentally established PLS colonies. Paperclips were used to attach excised portions of infested palmetto fronds (containing early instar PLS) to uninfested fronds.

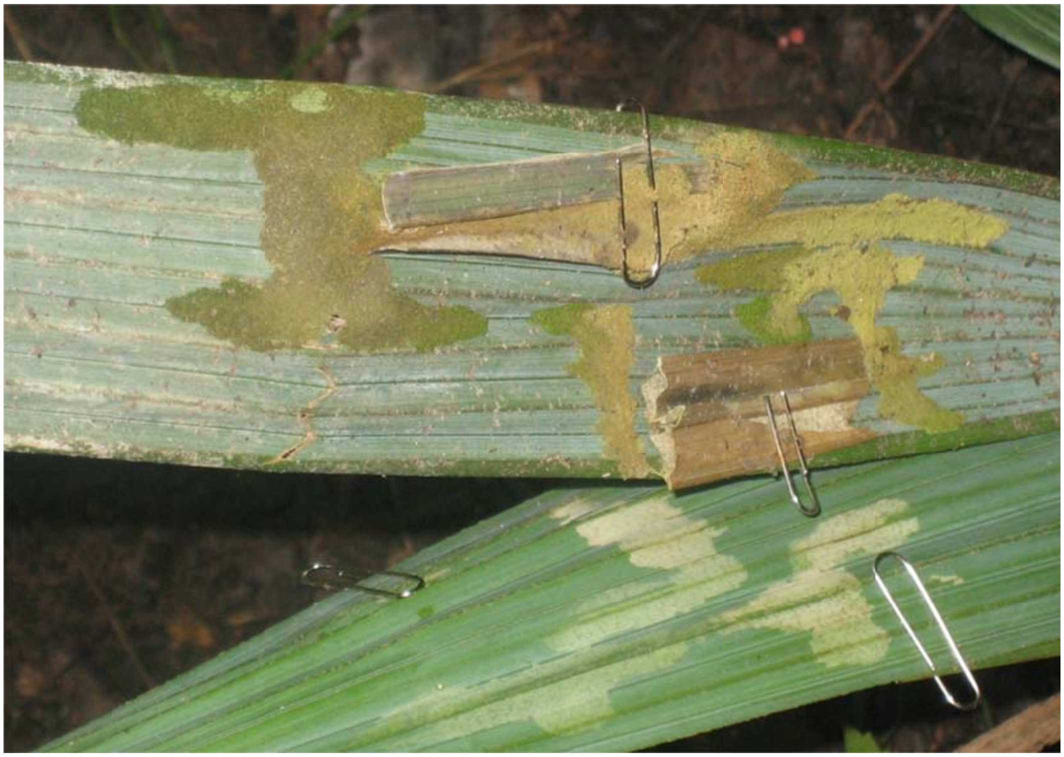

Supplement: Figure S1 — Photograph of experimentally established PLS colonies. Paperclips were used to attach excised portions of infested palmetto fronds (containing early instar PLS) to uninfested fronds. (PDF) [file pone.0022331.s001.pdf]
