# Supplementary material for: Spatial Ecology of the Palm-Leaf Skeletonizer, Homaledra sabelella (Lepidoptera: Coleophoridae)
Source: PLoS One. 2011 Jul 22;6(7):e22331. doi: 10.1371/journal.pone.0022331 (PMC3142117; doi:10.1371/journal.pone.0022331)
Supplement: Table S1 — Results from Poisson regression analyses for the effects of palmetto understory cover (bare, ferns, shrubs, other palmettos), soil wetness (dry, damp, wet), height (0–0.5 m, 0.5–1.5 m, 1.5–2.5 m, 2.5–3.5 m, >3.5 m) and isolation (low [Id≤0.1], medium [0.1<Id<1], high [Id>1]) and position (easting and northing) on the number of PLS pupae per palmetto. (PDF) [file pone.0022331.s002.pdf]

TABLE S1. Results from Poisson regression analyses for the effects of palmetto understory cover (bare, ferns, shrubs, other palmettos), soil wetness (dry, damp, wet), height (0-0.5 m, 0.5-1.5 m, 1.5-2.5 m, 2.5-3.5 m, >3.5 m) and isolation (low [$I_d \leq 0.1$], medium [$0.1 < I_d < 1$], high [$I_d > 1$]) and position (easting and northing) on the number of PLS pupae per palmetto.

ATV:

**Model**	**K**	**2LL**	R^2_M	$\%R^2_M$
full model*	15	4182.39	0.493	0.00
full model - all height effects	10	6736.08	0.180	63.44
full model - all Understory effects	10	5773.56	0.298	39.62
full model - all wetness effects	10	4670.57	0.432	12.33
full model - all isolation effects	10	5410.2	0.342	30.63
full model - all easting effects	10	5149.15	0.374	24.17
All main effects but no interactions	5	6720.64	0.181	63.30
full model - E*I	14	4287.84	0.480	2.66
full model - W*I	14	4425.53	0.463	6.07
full model - W*E	14	4224.77	0.488	1.10
full model - U*I	14	4785.06	0.419	14.96
full model - U*E	14	4333.03	0.475	3.78
full model - U*W	14	4227.58	0.487	1.17
full model - H*I	14	4353.81	0.472	4.29
full model - H*E	14	4525.81	0.451	8.55
full model - H*W	14	4479.33	0.457	7.40
full model - H*U	14	4556.38	0.447	9.30
Intercept	0	8193.65		

Note: Separate tests were performed for sites ATV and NAT.
* The full model is PLS = H U W E I H*U H*W H*E H*I U*W U*E U*I W*E W*I E*I; where PLS = number of palmetto leaf skeletonizer pupae, H = palmetto height, U = understory cover, W = soil wetness, I = Isolation, and E = easting. Northing was omitted from the table because all effects of northing had < 2% effect on PLS.

NAT:

Model	K	2LL	R^2_M	$\%R^2_M$
full model**	21	6875.13	0.543	0
full model - all height effects	15	10468.07	0.301	44.47
full model - all Understory effects	15	8668.28	0.422	22.27
full model - all wetness effects	15	7789.63	0.481	11.43
full model - all isolation effects	15	8482.79	0.434	19.98
full model - all easting effects	15	8960.05	0.402	25.87
full model - all northing effects	15	8134.76	0.458	15.69
all main effect but no interactions	6	11443.06	0.235	56.72
full model - E*I	20	7796.69	0.481	11.39
full model - W*I	20	6966.12	0.536	1.15
full model - W*E	20	6904.94	0.540	0.39
full model - U*I	20	7461.33	0.503	7.26
full model - U*E	20	6949.29	0.538	0.94
full model - U*W	20	6998.29	0.534	1.54
full model - H*I	20	7324.55	0.512	5.57
full model - H*E	20	7563.17	0.496	8.51
full model - H*W	20	7343.53	0.511	5.80
full model - H*U	20	7400.07	0.507	6.50
full model - N*U	20	6927.84	0.539	0.67
full model - N*W	20	6949.85	0.537	0.95
full model - N*E	20	6875.13	0.542	0.02
full model - N*I	20	7713.26	0.486	10.36
full model - N*H	20	7443.31	0.504	7.03
intercept	0	14939.65		

** The full model is PLS = H U W E I H*U H*W H*E H*I U*W U*E U*I W*E W*I E*I.
