# Supplementary material for: Spatial Ecology of the Palm-Leaf Skeletonizer, Homaledra sabelella (Lepidoptera: Coleophoridae)
Source: PLoS One. 2011 Jul 22;6(7):e22331. doi: 10.1371/journal.pone.0022331 (PMC3142117; doi:10.1371/journal.pone.0022331)
Supplement: File S1 — Supplement for Materials and Methods section. (PDF) [file pone.0022331.s003.pdf]

Supporting Information S1. Supplement for Materials and methods section.

Field survey of PLS. Sherburne Wildlife Management Area (WMA) is within the Mississippi River Valley and is dominated by hardwood species (*Nyssa sp.* [tupelo], *Liquidambar styraciflua* [sweetgum], *Platanus occidentalis* [Sycamore], *Quercus sp.* [oak], and *Taxodium sp.* [cypress]. The two research sites within Sherburne WMA, NAT and ATV were 4 km apart. The boundaries for each site were delineated by various natural and man-made landscape features (e.g., roads, streams, clear cuts) and natural breaks in the occurrence of *S. minor*. Palmettos at the periphery of the site were at least 300 m from any palmettos outside of the site.

The isolation index used in this study was derived by Hanski [1]. Here, $I = 1/[\Sigma\exp(^{-\alpha D})S]$; where S = PLS infestation size on a neighboring palmetto (i.e., number of pupae), D = distance (in meters) from the focal palmetto to the neighboring palmetto, and α = 1/average migration distance. The value of I is insensitive to the magnitude of α [1,2]. Therefore, I set $\alpha = 1$, a common convention in the literature [3].

Distribution of Palmettos. Morisita's index of dispersion was used to assess whether palmettos had a uniform, random or clumped distribution [4]. The index, I_d, was calculated in Passage 2.0 [5]. I_d has a chi-square distribution with n-1 degrees of freedom (where n=number of cells) and I used a χ^2 test to assess whether I_d was significantly different from zero [where $\chi^2 = I_d(\Sigma x - 1) + n - \Sigma x$; where x=count per cell). If I_d was significantly greater or less than one, the population was deemed clumped or uniformly distributed, respectively. Otherwise, if I_d was not significantly different from one, the population was considered randomly distributed.

PLS Spatial Patterns. The following statistical procedure was used to partition the effects of spatial structure (location or isolation) and the local environment (palmetto height, soil wetness, understory cover) on the distribution of PLS among palmettos at the two field sites. Spatial Eigenvector Mapping (SEVM) was used to encapsulate the spatial variation in the distribution of palmettos as a set of eigenvectors (i.e., spatial filters) [6,7]. In this analysis, geographic coordinates for palmettos were used to create a truncated distance or connectivity matrix, where the truncation distance was found by using a minimum-spanning tree algorithm [8]. The distance matrix was also centered so that the assumption of isotropy (i.e., spatial autocorrelation acts the same way in all directions) would be met [6]. Using the procedure outlined by Griffith & Peres-Neto [6], a topology-based eigenvector map was created from the centered connectivity matrix. The eigenvectors are mutually orthogonal and linearly uncorrelated map patterns and eigenvectors with positive or negative eigenvalues represent positive or negative spatial autocorrelation, respectively [6,9,10]. Small eigenvalues (in absolute value) indicate the absence of spatial autocorrelation and therefore the associated eigenvectors are uninformative regarding spatial structure. Among the n eigenvectors generated with this procedure (where n=number of locations [i.e., palmettos]), a forward selection procedure was used to narrow down the list of spatial descriptors. The procedure involves adding eigenvectors to the model (with PLS

abundance as the response variable) until the spatial autocorrelation in the residuals (measured by Moran's *I*) is below a given threshold, $I < 0.05$. This selection procedure was performed using Spatial Analysis in Macroecology SAM v4.0 [11].

Following the selection of spatial predictors, I evaluated the contributions of different spatial and environmental factors to PLS abundance using a generalized linear mixed model. Owing to counts with many zeros, PLS was Poisson distributed. Using Proc GLIMMIX in SAS 9.1 (Cary, NC, USA), this was accounted for in the model by defining the error term associated with PLS abundance as Poisson distributed. Isolation was treated as a categorical variable because the distribution of *I* was extremely right skewed and no transformation could normalize the data distribution. The three classes (with "high" being the most isolated) represented approximately equal thirds of the palmettos at each site. With the distance matrix centered and autocorrelation made isotropic, the spatial coordinate effects could reveal any directional trends in the data (i.e., an effect of prevailing winds or a road/edge effect).

McFadden's pseudo-R^2 (R^2_M) was used to evaluate to relative importance of each source of variation to predicting PLS abundance [12]. Here, R^2_M = 1- [(LL_{Full} – K)/$LL_{Intercept}$], where LL_{Full} and $LL_{Intercept}$ are the log likelihoods for the full and intercept-only models, respectively. *K* is the number of predictors in the model such that there is a cost to the goodness of fit as the number of variables in the model increases. R^2_M ranges between 0 and 1 but values tend to be lower than traditional R^2 values. R^2_M cannot be interpreted as an absolute measure of explained variance [13]. However, it can be used as an estimate of the improvement in the model relative to the null model (intercept only). It can also be used to estimate the percent contribution of a particular subset of model predictors to the goodness of fit (= %R^2_M). Here, %R^2_M = R^2_M (full) - R^2_M (subset)/ R^2_M (full). A high %R^2_M would indicate that the particular deleted variable(s) in the subset model made a large contribution to the goodness of fit of the full model.

Environmental determinants of PLS spatial structure. In the experiment to test whether the colonization of uninfested palmettos was dependent on palmetto height or understory cover, palmettos were excavated from locations nearby the ATV site and placed in 11 l pots. It was not practical to excavate and transport palmettos larger than 2.0 m tall. Therefore, the height treatment did represent the full range of palmetto sizes found in our field sites (some palmettos exceeded 3 m). Also, soil wetness was not included as a treatment for logistical reasons; i.e., number of excavated palmettos needed would have been too prohibitive.

Excavated palmettos were kept in pots and placed in the ground around the perimeters of five source patches, each consisting of 29-65 palmettos. The matrix within which each pot was placed consisted of either a pure shrub or open understory. Here, the matrix type occupied the intervening space between the source patch and planted palmetto, was ≥ 2 m wide, and completely surrounded the planted palmetto by at least 1 m. For consistency, only dry habitats were used. Because of the preponderance of a shrubby matrix around each source patch, I had to convert the matrix around some planted palmettos to an open matrix. In these instances (n = 22), the intervening vegetation was cut to the base with a machete and weed trimmer to simulate bare

ground. The simulated bare ground was assigned at random among the shrub-embedded planted palmettos to achieve the necessary number of open-understory replicates. For each source patch, I established an equal number of all combinations of the height-understory treatments.

Because only 38% of the experimental palmettos were colonized by moths, I elected to focus the analysis of these data on PLS colonization success, i.e., presence or absence of an active colony. Therefore, the effect of height, understory and their interaction on colonization success was evaluated with logistic regression. Source patch was also included as a block effect in the model to account for possible variation in colonization success. The analysis was performed using Proc GLIMMIX in SAS 9.1, with distribution=binomial in the model.

I conducted an additional test to evaluate whether the simulated bare ground differed from natural bare ground in its effects on PLS colonization success. Here, I used only palmettos in the naturally bare matrix and the simulated bare matrix. Height and source were also included in the logistic-regression model. Based on this analysis, I could find no evidence that the simulated bare ground was any different from natural bare ground in its effect on colonization probability (52% vs. 48%, respectively; $F_{1,50} = 0.5$, $P = 0.47$). These results validate the treatment of simulated and natural bare ground replicates as the same in the previous analysis (i.e., the effects of height and understory on colonization success).

In the PLS larval performance and pupal parasitism experiment, a fully crossed design involving height, understory cover and soil wetness was not possible because certain understory/wetness combinations were too rare (e.g., wet-shrub, or wet-fern). As a result, I used the following four understory-wetness categories: fern, shrub, open-dry and open-wet. For the open-wet understory, standing water would persist for days to several weeks following heavy rains. For all other understory habitats, standing water lasted < 1 d. Twenty replicates of all 12 height-understory combinations were used ($n = 240$ palmettos). To ensure independence among replicates, experimental palmettos were ≥ 8 m apart.

The source of early instar PLS larvae were cuttings of freshly infested sections of fronds from Sherburne WMA. To minimize variation in larval number and quality per cutting, I selected incipient infestations that were 5-10 cm long and 2-3 cm wide from 1-2 m tall palmettos in dry, shrubby habitats. Based on a subsample of cuttings, the number of larvae per cutting was 9.2 ± 1.1 ($n = 25$). Approximately 550 cuttings were made over a 2-d period. Two cuttings were attached with a paperclip to the underside of opposite fronds on each focal palmetto (Figure S1). This inoculum size (18.4 early instar larvae per palmetto), was meant to be relatively small so that intraspecific competition among larvae would not be substantial even in the smallest palmettos (but see Results).

After 4 d, palmettos were inspected for evidence of successful transfer of PLS caterpillars (the appearance of fresh feeding on the recipient frond adjacent to the attached cuttings). In cases where the transfer was unsuccessful (11.7% of all palmettos), the cutting was replaced with fresh

material.

The effects of understory cover/wetness and palmetto height on PLS performance measures and proportion parasitizied were assessed with separate two-way fixed factor ANCOVAs. To account for possible density effects, pupal number was included in the model as a covariate. Based on my findings that palmettos spatial heterogeneity was unimportant to the distribution of PLS (see Results), the spatial location of palmettos was not included as a factor in these models. The analyses were performed using SAS GLM and differences between treatment pairs were assessed with Tukey's HSD test. The distributions of the response variables were approximately normal with the exception of an excess of zeros for the proportion eclosed and an excess of zeros and ones for the proportion parasitized. Because the results of the analyses were qualitatively the same with or without these extreme values, I elected to retain them in the analyses. No transformations to the response variables were performed. To guard against an inflated type I error rate associated with four non-independent ANOVAs, I adjusted α (= 0.05) using a Bonferroni correction ($\alpha_{adj} = \alpha/4 = 0.0125$).

Dispersal limitation

The site used for the dispersal experiment was 3.5 km south of ATV and had no palmettos within a 0.7 km radius. The vegetation was similar to ATV and NAT. Potted palmettos were obtained from the colonization experiment. Potted palmettos were 1.5-2.0 m tall and all active PLS colonies were removed by hand prior to the start of the experiment.

Palmettos were positioned in concentric circles at fixed radii from an experimentally created source population of PLS (see below): 10 m (n=4), 25 m (8), 50 m (16), 100 m (24), and 200 m (43). Within each annulus, palmettos were spaced approximately evenly apart. Sample size increased with distance to increase the likelihood of colonization events at the outer distances.

To create the source of PLS, palmetto fronds infested with late-stage PLS pupae were harvested in late March, 2008 from ATV, NAT and other nearby sites (n = 170). Infested fronds were inserted upright into five 22 l buckets of water that were placed together in the center of the dispersal grid. Based on the dissection of twelve fronds, I estimated that 1474 PLS pupae comprised the source population.

For the second repetition of the dispersal experiment, the same site and potted palmettos were used. All potted palmettos were cleared of PLS infestations and reassigned at random to new locations within the grid. The source population for this repetition was 74 infested fronds with an estimated 838 PLS pupae. The same procedure was followed as described for the previous release.

For both replicates of this experiment, the mean number of colonies per potted palmetto at

each distance d was computed (C_d). To make the second trial comparable to the first, I multiplied C_d by 1.75 to account for the smaller source population in the second trial (1474/838).

Two phenomenological dispersal models were fit to the colonization-with-distance data, the negative exponential function (NEF) and the inverse power function (IPF) [14]. For the NEF, $C_d = ae^{-bd}$ and for the IPF, $C_d = ad^{-b}$. a and b are parameters to be estimated. The fit of both distributions to the dispersal data were evaluated with least-squares regression and goodness of fit was determined from the model R^2. To increase statistical power, data from both trials were pooled. Using the best-fit model, I performed a Monte Carlo simulation to estimate the range of PLS dispersal. The slope, intercept and associated standard deviations were used to generate 1,000 distributions of PLS colonies (truncated at 1,000 m). From these simulated distributions, I estimated the median dispersal distance and the distance that contained 95% of the colonies and their 95% confidence intervals. The simulation was performed using Matlab v7.11.

Literature Cited

1. Hanski I (1994) A practical model of metapopulation dynamics. Journal of Animal Ecology 63: 151-162.
2. Hanski I (1999) Metapopulation ecology. New York: Oxford University Press.
3. Prugh LR (2009) An evaluation of patch connectivity measures. Ecological Applications 19: 1300-1310.
4. Krebs CJ (1999) Ecological Methodology. Menlo Park, CA: Benjamin/Cummings.
5. Rosenberg MS, Anderson CD (2011) Pattern Analysis, Spatial Statistics and Geographic Exegesis. Version 2. Methods in Ecology and Evolution 2.
6. Griffith DA, Peres-Neto PR (2006) Spatial modeling in ecology: the flexibility of eigenfunction spatial analyses. Ecology 87: 2603-2613.
7. Dormann CF, Mcpherson JM, Araujo MB, Bivand R, Bolliger J, et al. (2007) Methods to account for spatial autocorrelation in the analysis of species distributional data: a review. Ecography 30: 609-628.
8. Legendre P, Legendre L (1998) Numerical Ecology. New York, New York: Elsevier.
9. Griffith DA (2000) Eigenfunction properties and approximations of selected incidence matrices employed in spatial analyses. Linear Algebra and Its Applications 321: 95-112.
10. Borcard D, Legendre P (2002) All-scale spatial analysis of ecological data by means of principal coordinates of neighbour matrices. Ecological Modelling 153: 51-68.
11. Rangel TF, Diniz JAF, Bini LM (2010) SAM: a comprehensive application for Spatial Analysis in Macroecology. Ecography 33: 46-50.
12. Nagelkerke NJD (1991) A note on a general definition of the coefficient of determination. Biometrika 78: 691-692.
13. Long JS (1997) Regression models for categorical and limited dependent variables. Thousand Oaks, California: Sage Publications.
14. Turchin P (1998) Quantitative analysis of movement: measuring and modeling population redistribution in animals and plants. Sunderland, Massachussetts: Sinauer Associates.
